# Validation of the Amharic Version of the Full and Short Form of the Trust in Oncologist Scale Among Cervical Cancer Patient

**DOI:** 10.1002/cnr2.70718

**Published:** 2026-10-12

**Authors:** Sosina W. Tilahun, Adiam Nega, Lealem Wagaw, Marij A. Hillen, Adamu Addissie

**Affiliations:** ^1^ Department of Emergency, Critical, and Chronic Care Nursing School of Nursing and Midwifery, College of Health Sciences, Addis Ababa University Addis Ababa Ethiopia; ^2^ Department of Health Systems Management and Health Policy School of Public Health, College of Health Sciences, Addis Ababa University Addis Ababa Ethiopia; ^3^ Department of Epidemiology and Biostatistics School of Public Health, College of Health Sciences, Addis Ababa University Addis Ababa Ethiopia; ^4^ Department of Medical Psychology, Amsterdam Public Health Research Institute University of Amsterdam, Amsterdam UMC Amsterdam the Netherlands

**Keywords:** Amharic version, cervical cancer, short‐form scale, Trust in Oncologist Scale, validation

## Abstract

**Background:**

The Trust in Oncologist Scale (TiOS) is a tool used to measure cancer patients' self‐reported trust in their oncologists. It has been validated in Dutch, English, Japanese, and Italian.

**Aims:**

This study aims to validate the Amharic version of the tool and develop its short form.

**Methods and Results:**

A cross‐sectional study design was employed among 197 cervical cancer patients at a tertiary teaching hospital in Ethiopia (February to April 2025). Internal consistency, convergent validity, and the structural validity of both the full scale and short form of the Amharic TiOS were tested. The Amharic TiOS mean score was 69.95 (±9.41, range 34–90), with a mean score of 3.89 (±0.52). Neither the four‐factor nor the one‐factor models fit the full scale. A unidimensional five‐item solution emerged with two possible item sets: (1, 6, 15, 14, and 18) and (1, 3, 15, 14, and 18). To preserve content validity, the second set was selected. This five‐item scale yielded a mean score of 4.01 (±0.57), strong internal consistency (*α* = 0.88), and significant correlations with time since diagnosis (*r* = 0.20, *p* < 0.05), perceived levels of shared decision‐making (*r* = 0.38, *p* < 0.001), and health literacy (*r* = −0.32, *p* < 0.01).

**Conclusion:**

Unlike the full Amharic scale, the short form demonstrated acceptable psychometric properties and can be used to assess trust in both clinical practice and research settings among cervical cancer patients in Ethiopia.

## 1 Background

Trust is vital for fostering interpersonal relationships and community cohesion, highlighting its significance for human coexistence. Its definition encompasses aspects of vulnerability, making it essential for a therapeutic relationship between patient and physician, particularly, in the context of serious illness [1]. The physician–patient relationship is often described as *asymmetrical*, characterized by an imbalance of knowledge and power [2]. A study drawing on Meyer's interpersonal theory notes that patients need to acknowledge their vulnerability, including recognition of their emotional and physical dependence on providers for their health outcomes [3]. Consequently, trust is regarded as the heart of a successful doctor–patient relationship in clinical care [4].

Trust is essential in cancer care due to the complexity of the disease and the uncertainties associated with the treatment process [5]. This complexity necessitates patients' trust in their oncologist, given their dependency not only on medical expertise but also on guidance, reassurance, and decision‐making [5, 6]. Increased levels of trust in the oncologist have been associated with greater involvement in treatment decision‐making and reduced fear, highlighting trust as an ethical imperative in oncology care [7], as well as improved treatment adherence, which in turn decreases loss to follow‐up [8]. Conversely, medical mistrust has been linked to adverse outcomes, including unwillingness to participate in clinical research, reduced use of preventive services, lower patient satisfaction and treatment adherence, and poorer overall physical and mental health [9].

The assessment of trust using validated tools is important in empirical application. Several tools have been developed to measure patients' trust in their doctors, primarily within primary health care settings [10, 11, 12, 13]. Most of these tools are unidimensional in nature and employ a 5‐point Likert scale. Among these tools, the Trust in Oncologist Scale (TiOS), developed in Dutch based on the Physician Trust Scale [14] and findings from a qualitative study on oncology patients [15], is the only tool specifically designed to measure cancer patients' trust in their oncologists.

Theoretically, the TiOS incorporates four dimensions: fidelity, competence, honesty, and caring. Both the Dutch version and the subsequently developed English version have demonstrated good psychometric properties [14, 16]. Nevertheless, in empirical applications, the one‐factor model has proven more practical. Short‐form, five‐item, unidimensional versions of the TiOS have since been developed in English, Japanese, Italian, and Dutch versions [16, 17, 18, 19], with initial evidence supporting their reliability and confirming the mono‐factorial structure. To further ensure the cross‐cultural validity of the TiOS and strengthen its relevance, the structure and psychometric properties of the tool must be validated in diverse socio‐cultural contexts.

Ethiopia faces a rising incidence of cervical cancer, which is a leading cause of cancer‐related death among women [20, 21, 22, 23] and it compromises health‐related quality of life through both the disease burden and its treatment effects [24]. Evidence shows that trust in oncologists is an important determinant of treatment adherence, coping, and psychosocial well‐being among cancer patients [25, 26]. Therefore, validating TiOS in the Amharic, the most widely spoken national language of the country, is essential. Furthermore, such validation would strengthen the patient‐centered approach to cervical cancer care within Ethiopia's sociocultural context.

Therefore, this study primarily aims to investigate dimensionality, internal consistency, convergent validity, and the structural validity of the full‐scale Amharic TiOS (AM‐TiOS). Second, it aims to develop and validate a short‐form Amharic TiOS (AM‐TiOS‐SF) in a sample of Ethiopian cervical cancer patients.

## 2 Methods

### 2.1 Design

This study employed a cross‐sectional study design. First, it translated the TiOS from English to Amharic. Second, it validated the AM‐TiOS; and finally, it developed and validated the AM‐TiOS‐SF.

### 2.2 Translation of the TiOS Full Scale From the English Into Amharic

The adaptation of the TiOS to Amharic followed a structured process. Two independent bilingual experts, fluent in English and Amharic and familiar with medical terminology, conducted the forward translations. Clinical oncologists reviewed these translations to ensure conceptual equivalence and contextual relevance. An independent translator, blinded to the original tool, performed a back translation into English. Discrepancies were resolved through systematic discussion by an expert panel comprising the translators, oncologists, and principal investigator, with decisions documented. Emphasis was placed on linguistic accuracy, cultural sensitivity, and preservation of the tool's constructs: competency, honesty, fidelity, caring, and global items. The process adhered to the ITC Guidelines for Translating and Adapting Tests [27].

### 2.3 Validation of the AM‐TiOS and Development and Validation of the AM‐TiOS‐SF

#### 2.3.1 Participants

Women with confirmed cervical cancer were consecutively recruited from Tikur Anbessa Specialized Hospital (TASH), Addis Ababa, Ethiopia, between February and April 2025. TASH was selected, as it is one of the country's largest tertiary teaching hospitals and the pioneer in radiation therapy. Its high patient volume ensures access to a diverse population from across the country, representing varied sociodemographic and clinical characteristics.

An oncology nurse from another hospital authorized by TASH to assist with introducing women with confirmed cervical cancer to the study. To minimize coercion or perceived clinical obligation, the nurse had no treatment responsibilities at TASH and no clinical relationship with the participants. The nurse's role was to identify eligible participants and inform them about the study. Permission was obtained before any contact details were transferred, and only women who voluntarily expressed interest were contacted by the research team. This arrangement was included in the proposal that was approved by the Research Ethics Committee of the Department of Epidemiology and Biostatistics, School of Public Health, Addis Ababa University.

The research team later contacted interested candidates, confirming their voluntariness and eligibility. Inclusion criteria required at least a consultation with an oncologist, one round of adjuvant or neoadjuvant chemotherapy, no clinically diagnosed mental disorders checking their medical records, and fluency in Amharic for communication with their oncologist. In total, 203 women enrolled; 197 completed the questionnaires, while 4 provided incomplete responses and 2 declined participation during the actual data collection.

#### 2.3.2 Data Collection Process and Tools

Interviewer‐administered questionnaires were used at the outpatient department of the cancer center of TASH. The interviews were conducted in a private consultation room to minimize social desirability bias. Participants were clearly informed that their responses would not be shared with their oncologists. Data collectors were trained oncology nurses from another institution, reducing the risk of role‐related influence. They were trained on uniformity in instructions, presenting the same response option in the same order, and avoiding leading prompts. For participants with limited literacy, the data collectors were trained to support them to read the questions aloud and clarify response options without offering interpretations and suggestions to answer. The questionnaires were administered in the following order:

##### 2.3.2.1 Sociodemographic and Clinical Information

Data on sociodemographic factors (age, residence, religion, education, occupation, income, marital status, and source of treatment expenses) and clinical characteristics (disease stage, time since diagnosis, Eastern Cooperative Oncology Group [ECOG] Performance Status Scale, and consultation rounds) were adapted from prior literature [28, 29, 30, 31, 32, 33].

##### 2.3.2.2 Self‐Reported Questionnaires

Participants completed the AM‐TiOS, the shared decision‐making questionnaire (SDM‐9), and the Health Literacy Scale (HLS‐Q).

###### 2.3.2.2.1 The AM‐TiOS

The AM‐TiOS is an 18‐item self‐report tool covering four dimensions of trust: competence (Items 1, 6, 9, 11), honesty (Items 2, 3, 12), fidelity (Items 4, 5, 8, 10, 15), caring (Items 7, 13, 14, 16), plus two global items (17, 18). Reponses use a 5‐point Likert scale (1 = *strongly disagree* to 5 = *strongly agree*). Negatively keyed items (9, 11, and 13) were reverse‐scored [14].

###### 2.3.2.2.2 The Amharic Shared‐Decision Making (SDM‐9)

The SDM‐9 is a 9‐item tool developed in 2010 to measure patients' perceived involvement in care using a 6‐point Likert scale (0 = *completely disagree* to 5 = *completely agree*). Higher scores reflect greater shared decision‐making (SDM). The tool has shown excellent internal consistency (*α* > 0.92) in both German [34] and the United Kingdom studies [35], and in the current study (*α* = 0.98).

###### 2.3.2.2.3 The Amharic HLS‐Q

The HLS‐Q, developed by the European Health Literacy Consortium, is a 47‐item tool assessing health promotion (16 items), disease prevention (15 items), and healthcare (16 items) [36]. Responses use a 4‐point scale (1 = *extremely difficult* to 4 = *very easy*), with higher scores indicating greater literacy. The tool demonstrated excellent internal consistency (*α* > 0.90) and validity across multiple countries [37, 38] and excellent internal consistency in the current study (*α* = 0.98).

The adaptation of the HLS‐Q and SDM‐9 instruments into Amharic involved a multistep process. Two independent experts, fluent in both English and Amharic and familiar with the medical terminologies, conducted the translations. These translations were then reviewed by a clinical oncologist and the principal investigators. An expert, blinded to the original tools, performed a back‐translation to the original language of English. Any discrepancies between the back‐translated and original instruments were resolved through discussion. The final Amharic versions of HLS‐Q and SDM showed excellent internal consistency, with Cronbach's alphas of 0.98 and 0.98, respectively.

#### 2.3.3 Data Analysis

Descriptive statistics summarized sociodemographic and clinical variables as means (SDs) and frequencies (proportions). Based on psychometric properties (factor loadings, item–scale correlations, item correlations, and variability), a AM‐TiOS‐SF was developed. To ensure transparency and reproducibility, item selection followed a pre‐specified sequential statistical criteria, beginning with factor loadings, followed by item‐scale, inter‐item correlations, and item variability. First, items with factor loadings ≥ 0.40, item–scale correlations ≥ 0.50, and inter‐item correlations between 0.30 and 0.70 were retained to avoid redundancy and weak associations. Item variability was ensured by standard deviation at SD > 0.50 [39, 40, 41]. Cultural appropriateness was ensured by a panel of bilingual experts, clinical oncologists, and an independent translator.

Confirmatory factor analysis (CFA) was conducted in STATA‐17 testing the original four‐factor and unidimensional models by using maximum likelihood (ML) estimation with Pearson correlations [42]. Because the items of TiOS are skewed, robust *χ* ^2^ statistics (Satorra–Bentler correction) were applied to adjust for non‐normality. This adjustment reduced inflation of the *χ* ^2^ test under non‐normal conditions. ML was used due to software constraints, and missing data were minimal (< 3%) and handled through listwise deletion [43]. Factor identification was achieved by fixing one loading per factor, and residual correlations were not permitted.

Model fit was indicated by a nonsignificant *χ* ^2^, a root mean square error of approximation (RMSEA) < 0.06, a standardized root mean square residual (SRMR) < 0.08, a comparative fit index (CFI) ⁓0.95, and a goodness of fit index (GFI) > 0.90 [44]. To preserve content validity, one item from each construct was retained, ensuring the AM‐TiOS‐SF was statistically optimized and culturally appropriate. Finally, as this was the first validation of TiOS in Amharic, convergent validity was assessed exploratorily, with no prior hypothesis regarding its correlation with SDM‐9 and HLS‐Q [45] along with time since diagnosis.

## 3 Results

### 3.1 Sample Characteristics of the Participants

Of 203 patients approached, 197 participated (97%). The mean age was 54.44 ± 10.41 years. Most had not attended formal education (109; 55.3%) and were unemployed (148; 75.1%). Nearly two‐thirds were married (126; 63.9%) and lived in urban areas (124; 62.9%). The majority received services through health insurance (176; 89.3%).

Most participants were diagnosed with Stage III (92; 46.7%). Over half reported an ECOG performance status of II (122; 61.9%). On average, they had 16.27 ± 10.88 consultations with oncologists, and the mean time since diagnosis was 34 ± 38.12 months.

Participants had a mean SDM score of 24.94 ± 9.12 (range 7–44; mean score 2.77). The mean health literacy score was 114.37 ± 25.98 (range 75–188; mean score 2.43). Sample characteristics are presented in Table 1.

**TABLE 1:** Sample characteristics of women with cervical cancer, from February to April 2025 (*n* = 197).

Sociodemographic characteristics	Frequency	Percentage
Age in years		
< 50 years old	71	36.00
≥ 50 years old	126	64.00
Mean (SD)	54.44 (±10.41)	
Area of residence		
Addis Ababa	124	62.90
Nonresident of Addis Ababa	73	37.10
Religion affiliation		
Orthodox	143	72.60
Protestant	21	10.70
Muslim	32	16.20
Other^a^	1	0.50
Educational status		
Did not join formal education	109	55.30
< High school	56	28.40
≥ High school	32	16.30
Occupation		
Unemployed	148	75.10
Employed	49	24.90
Average monthly income in ETB mean (SD)	3015.07 (±2489.10)	
Marital status		
Never been married (Single)	14	7.20
Married	126	63.90
Separated	57	28.90
Source of treatment expense		
Health insurance	176	89.30
Self‐sponsored	21	10.70
Stage of the disease		
Stage I	6	3.00
Stage II	53	26.90
Stage III	92	46.70
Stage IV	46	23.40
Time since diagnosis in months mean (SD)	34.25 (±38.12)	
Eastern cooperative oncology group (ECOG)		
I	43	21.80
II	122	61.90
III	28	14.20
IV	4	2.00
Round of consultation mean (SD)	16.27 (±10.88)	
Shared decision‐making (SDM)	24.94 (±9.12)	
Health literacy score	114.36 (±25.10)	

Abbreviations: ECOG, Eastern Cooperative Oncology Group; ETB: Ethiopian Birr; Rural: those who are living out of Addis Ababa city; SD, standard deviation; SDM, shared decision making; Urban residents: those living in Addis Ababa.

^a^ Jehovah witness.

### 3.2 Psychometric Properties of the AM‐TiOS

#### 3.2.1 Four‐Factor Model Results

Table 2 presents the Amharic full‐scale items of the TiOS. Skewness ranged from −1.55 to −0.58. The mean trust score across 18 items was 3.89 ± 0.52 (range 1–5). Internal consistency was excellent (Cronbach's *α* = 0.92), with item–scale correlations between 0.30 and 0.82.

**TABLE 2:** Overview of all items of the Am‐TiOS full scale, including their mean, standard deviation (SD), skewness and item‐scale correlation (*n* = 197).

Item	content	Mean	SD	Skewness	Item‐scale correlation
1.	Your doctor is very careful and precise (ዶክተርዎ በጣም ጠንቃቃ እና ትክክለኛ ነው)	4.20	0.62	−0.68	0.68
2.	Your doctor is totally honest in telling you about all the different treatment options available for your condition (ዶክተርዎ ለህመምዎ ስላሉት የተለያዩ የሕክምና አማራጮች ሲነግርዎ ሙሉ በሙሉ ታማኝ ነው)	3.89	0.90	−0.98	0.70
3.	Your doctor always gives you honest information about your prospects (ዶክተርዎ ሁል ጊዜ ስለ ተስፋዎ ትክክለኛ መረጃ ይሰጥዎታል)	3.99	0.79	−0.88	0.76
4.	Your doctor cares a lot about your health (ዶክተርዎ ስለ ጤንነትዎ በጣም ያስባል)	4.24	0.62	−0.60	0.62
5.	Your doctor always tells you everything you want to know about your illness (ዶክተርዎ ስለ ህመምዎ ማወቅ የሚያስፈልግዎትን ሁሉ ሁልጊዜ ይነግርዎታል)	3.61	0.97	−0.60	0.74
6.	You think your doctor can handle any health problem, even a very serious one (ዶክተርዎ ማንኛውንም የጤና ችግር ማከም ይችላል ብለው ያስባሉ፣ በጣም ከባድ የሆነውን እንኳን)^a^	3.85	0.77	−1.03	0.69
7.	Your doctor always takes his/her time with you (ዶክተርዎ ሁል ጊዜ ከእርስዎ ጋር ጊዜ ይወስዳል)	3.80	0.81	−0.78	0.74
8.	Your doctor explains everything so that you can consent to medical decisions (ዶክተርዎ ሁሉንም ነገር ያብራራልዎታል፣ ይህም የሕክምና ውሳኔዎችን አንድወስኑ ያስችልዎታል)	3.50	0.99	−0.58	0.56
9.^a^	Sometimes you worry that your doctor's medical decisions are wrong (አንዳንድ ጊዜ የዶክተርዎ የሕክምና ውሳኔዎች የተሳሳተ ነው ብለው ይጨነቃሉ)	4.21	0.76	−1.55	0.30
10.	Your doctor only thinks about what is best for you (ዶክተርዎ ስለሚጠቅመዎ ነገር ብቻ ነው የሚያስብ)	3.89	0.66	−1.12	0.45
11.^a^	Sometimes your doctor does not pay full attention to what you want to tell him/her (አንዳንድ ጊዜ፣ ዶክተርዎ ለእሱ/እሷ ልትነግሩት ለምትፈልጉት ነገር ሙሉ ትኩረት አይሰጥዎትም)^a^	3.91	0.83	−1.26	0.55
12.	Your doctor will always tell you the truth about your health, even if it's bad news (ምንም እንኳን መጥፎ ዜና ቢኖርም ዶክተርዎ ስለ ጤናዎ ሁልጊዜ እውነቱን ይነግርዎታል)^a^	3.93	0.74	−1.04	0.60
13.^a^	You have doubts whether your doctor really cares about you as a person (ዶክተርዎ በእርግጥ እንደ ሰው ያስባልዎት እንደሆነ ጥርጣሬዎች አሉዎት)	3.55	0.81	−1.27	0.40
14.	Your doctor listens with care and concern to all the problems you have (ዶክተርዎ ያለዎትን ችግሮች ሁሉ በጥንቃቄ እና በአሳቢነት ያዳምጣል)	3.95	0.65	−1.14	0.64
15.	Your doctor will do whatever it takes to get you all the care you need (ዶክተርዎ የሚያስፈልግዎትን እንክብካቤ ሁሉ ለማግኘት የሚያስፈልገውን ሁሉ ያደርጋል)	4.02	0.73	−0.94	0.71
16.	Your doctor is available for you whenever you need him/her (ዶክተርዎ በሚያስፈልግዎት ጊዜ ሁሉ ለእርስዎ ዝግጁ ነው/ትሆናለች)	3.87	0.90	−0.80	0.57
17.	You have no worries about putting your life in your doctor's hands (ህይወትህን በዶክተርህ እጅ ለማስቀመጥ ምንም አይነት ስጋት የለብህም)	3.62	0.73	−1.13	0.75
18.	All in all, you have complete trust in your doctor (በአጠቃላይ፣ በዶክተርዎ ላይ ሙሉ እምነት አለዎት)	3.92	0.66	−0.78	0.82

^a^ Reversed item.

CFA of the four‐factor model showed poor fit: *χ* ^2^ = 526.27 (df = 125, *p* = 0.000), RMSEA = 0.128, SRMR = 0.084, and CFI = 0.82, all failing to reach acceptable cutoff levels. This was not due to high collinearity, as interitem correlations were moderate (47.1% between 0.40 and 0.60, 39.9% < 0.40, and 13.1% > 0.60) (Table 3).

**TABLE 3:** Inter‐item correlation for the full‐scale AM‐TiOS.

Item	1	2	3	4	5	6	7	8	9	10	11	12	13	14	15	16	17	18
1	1.00																	
2	0.49	1.00																
3.	0.59	0.74	1.00															
4.	0.54	0.36	0.55	1.00														
5.	0.44	0.72	0.71	0.47	1.00													
6.	0.47	0.51	0.58	0.50	0.57	1.00												
7.	0.50	0.51	0.55	0.48	0.65	0.59	1.00											
8.	0.30	0.61	0.53	0.33	0.71	0.34	0.59	1.00										
9.	0.12	0.17	0.18	0.24	0.12	0.29	0.13	0.03	1.00									
10.	0.48	0.22	0.31	0.45	0.24	0.33	0.33	0.11	0.14	1.00								
11.	0.36	0.44	0.44	0.28	0.39	0.33	0.40	0.27	0.49	0.21	1.00							
12.	0.46	0.60	0.57	0.40	0.51	0.46	0.39	0.42	0.16	0.29	0.33	1.00						
13.	0.45	0.14	0.23	0.34	0.13	0.21	0.29	0.01	0.49	0.39	0.51	0.15	1.00					
14.	0.57	0.40	0.51	0.44	0.48	0.51	0.61	0.38	0.14	0.42	0.36	0.40	0.31	1.00				
15.	0.58	0.39	0.54	0.49	0.49	0.56	0.66	0.39	0.21	0.30	0.36	0.36	0.36	0.63	1.00			
16.	0.34	0.53	0.42	0.38	0.58	0.39	0.41	0.51	0.17	0.22	0.28	0.44	0.65	0.45	0.39	1.00		
17.	0.54	0.49	0.54	0.45	0.49	0.59	0.63	0.35	0.28	0.42	0.40	0.45	0.37	0.65	0.65	0.47	1.00	
18.	0.63	0.66	0.60	0.53	0.54	0.61	0.67	0.40	0.26	0.49	0.45	0.49	0.43	0.69	0.73	0.47	0.88	1.00

#### 3.2.2 One‐Factor Model Results

The CFA of the one‐factor model showed poor fit. The likelihood ratio test against the saturated model was significant (*χ* ^2^ (135) = 727, *p* < 0.000), indicating the model did not reproduce the data. Fit indices were below thresholds (RMSEA = 0.149, CFI = 0.740), though SRMR was near acceptable (0.088). The coefficient of determination was high (CD = 0.949), suggesting substantial explained variance. Standardized item loadings ranged from 0.47 to 0.85.

The mean score of the full AM‐TiOS correlated positively with time since diagnosis (*r* = 0.17, *p* < 0.050) and perceived SDM (*r* = 0.50, *p* < 0.001) and negatively with health literacy score (*r* = −0.43, *p* < 0.010). Consultations with oncologists showed no significant correlation.

### 3.3 Psychometric Properties of the AM‐TiOS‐SF

The CFA supported a unidimensional five‐item solution with two alternative item combinations: (1, 6, 14, 15, and 18) and (1, 3, 14, 15, and 18).

*The first model* (items 1, 6, 14, 15, 18) showed an excellent fit: mean trust 3.98 ± 0.57 (range 1–5); *χ* ^2^ (5) = 1.513, *p* = 0.912; RMSEA = 0.000 (90% CI: 0.000–0.038, *p*‐close = 0.967); CFI = 1.000 and TLI = 1.00; SRMR = 0.009; CD = 0.902. Information criteria were AIC = 1555.91 and BIC = 1605.15, further supporting the adequacy of the model. Residual variances ranged between 0.10 and 0.32, suggesting that the latent factor explained a substantial proportion of item variance. Internal consistency was strong (Cronbach's *α* = 0.88), with item‐scale correlations ranging from 0.47 to 0.73.

*The second model* (Items 1, 3, 14, 15, 18) also suggested acceptable fit: mean trust 4.01 ± 0.57 (range 1–5) *χ* ^2^ (5) = 9.30, *p* = 0.098; RMSEA = 0.066 (90% CI: 0.000–0.131, *p*‐close = 0.285); SRMR = 0.022; CFI = 0.992; TLI = 0.983; CD = 0.900. Information criteria were AIC = 1562.76 and BIC = 1612.01, supporting the relative adequacy of the selected model compared with alternatives. Residual variances ranged between 0.10 and 0.32, suggesting that the latent factor explained a substantial proportion of item variance. Internal consistency was strong (Cronbach's *α* = 0.88), with item‐scale correlations ranging from 0.64 to 0.81.

Although the first model demonstrated a better statistical fit, to maintain content validity, the second model was selected, as it included one item from each construct. To further strengthen content validity, bilingual experts, clinical oncologist, and independent translator (lay man to the clinical sciences) reviewed the items for conceptual relevance and cultural appropriateness, confirming that the items were comprehensible and representative of the intended constructs in our context (Table 4).

**TABLE 4:** Overview of items 1, 3, 14, 15, and 18 of the AM‐TiOS‐SF, including item‐scale correlation (*n* = 197).

Item	Content	Inter‐item correlation	Factor loading in EFA	Dimension	Mean (SD)	Skewness
1 (1)	Your doctor is very precise and careful	0.66	0.81	Competency	4.20 (±0.62)	−0.68
3 (2)	Your doctor always gives you honest information about your prospects	0.79	0.78	Honesty	3.99 (±0.79)	−0.88
14 (3)	Your doctors listens with care and concern to every problem you have	0.71	0.82	Caring	3.91 (±0.65)	−1.14
15 (4)	Your doctor will do whatever it takes to get you all the care you need	0.76	0.84	Fidelity	3.93 (±0.73)	−0.94
18 (5)	All in all, you have complete trust in your doctor	0.81	0.89	Global Items	4.02 (±0.66)	−0.78

*Note:* Mean scores on both models of AM‐TiOS‐SF correlated positively with time since diagnosis (*r* = 0.20, *p* < 0.05) and shared decision‐making (*r* = 0.38, *p* < 0.001) and negatively with health literacy (*r* = −0.32, *p* < 0.01).

## 4 Discussion

This study aimed to validate the full‐scale AM‐TiOS and to develop the AM‐TiOS‐SF. The study was conducted in accordance with COnsensus‐based Standards for the selection of health Measurement Instruments (COSMIN) [46]. We assessed content validity through translation and expert review, structural validity through CFA, internal consistency through Cronbach's *α*, and construct validity through correlation with SDM‐9, health literacy, and time since diagnosis.

Consequently, neither the four‐factor nor one‐factor structures of the full scale adequately fit the data. This poor fit may be attributed to several interrelated factors. Sample characteristics of low‐literacy level and heavy disease burden may have impacted the response pattern and reduced reliability of negatively worded items [47]. Moreover, the response style of collectivist culture, which is extreme responding, might bias the Likert Scale and obscure multidimensional characteristics of the tool [48]. Thus, the constructs of competency, honesty, caring, and fidelity may overlap strongly, indicating the importance of local validation rather than assuming the generalizability of the original factor structure.

Following CFA, two short‐form versions of AM‐TiOS emerged, with the second (Items 1, 3, 14, 15, 18) selected to preserve content validity. This aligns with validations in Italy (Items 2, 6, 14, 15, 18) [18], the Netherlands (Items 2, 6, 14, 15, 18) [19], and Japan (Items 11, 3, 14, 4, 18) [17], all of which confirmed five‐item TiOS‐SF versions. The Ethiopian's selection closely parallels Italy and the Netherlands but differs by prioritizing item 1 (*Your doctor is very careful and precise*) over item 2 (*Your doctor is totally honest in telling you about all the different treatment options available for your condition*) and substituting item 6 for 3 to maintain validity. The difference reflects contextual variations in how patients construct trust in oncology care, with expectations of honesty versus precision shaped by sociocultural norms [6]. A recent bioethics consortium report further emphasized that oncology care must balance autonomy, beneficence, and truth‐telling and that cultural competency is essential, as patient expectations of honesty and precision vary among low‐ and high‐income countries [49].

Unlike previous studies validated among general cancer patients [17, 18, 19], this study developed and validated a five‐item version among cervical cancer patients. The findings demonstrate that TiOS‐SF is both culturally adaptable and responsive to disease‐specific contexts. Cervical cancer patients face distinct psychosocial challenges, including stigma [21] and reproductive health concerns [24]. By validating the tool among this population, the study extends evidence beyond general oncology, reinforcing its utility in gynecologic oncology and supporting broader cross‐context applicability.

This study indicates that the TiOS‐SF is a reliable and valid measure of cervical cancer patients' trust in their oncologist. The five items capture all five dimensions of the full‐scale AM‐TiOS: competence, honesty, fidelity, caring, and global trust. Together, they form a coherent one‐dimensional scale, with structural validity. These findings show that trust can be unified into a single factor expressed through multiple facets. Consistent with validations in Italy [18], the Netherlands [19], and Japan [17], the five‐item short form retained the psychometric strength of the full 18‐item scale. The comparability of results across these countries and Ethiopia provides cross‐cultural evidence that the TiOS is a robust and adaptable tool.

Convergent validity was confirmed by a moderate positive correlation between AM‐TiOS‐SF and time since diagnosis, a finding that is both theoretically and clinically meaningful. Trust dynamics develop over time and may reflect both confidence in the oncologist and patient vulnerability during periods of uncertainty [6, 50]. Cervical cancer, as a chronic illness that requires prolonged treatment, heightens patients' vulnerability [3]. Although the round of consultation did not correlate with the AM‐TiOS‐SF, repeated encounters may allow doctors to demonstrate competence, fidelity, honesty, and caring, thereby reinforcing trust over time. This result is consistent with studies in Italy [18] and the Netherlands [19]. Beyond scale validation, literature confirms that trust deepens as patients navigate complex treatment processes [51], which is the case for cervical cancer.

The study found a positive moderate correlation between AM‐TiOS‐SF and patients' perceived SDM, shedding light to patient participation. Trust is closely tied to communication quality and involvement in decision‐making [4]. SDM fosters trust by prioritizing patients' values and preferences [52] and allows oncologists to demonstrate fidelity and caring [53]. Thus, the correlation supports the scale's convergent validity and highlights its sensitivity to relational and ethical dimensions of cancer care.

On the contrary, a negative correlation between Am‐TiOS‐SF and health literacy score highlights a complex patient‐doctor dynamic. Patients with low literacy may rely more on their oncologist, placing greater trust as a coping mechanism when vulnerable or unable to evaluate medical information [3]. This can be understood as blind trust, placing complete trust in someone without any question [4], shaped by limited health literacy and cultural practice [6] or hierarchical communication [2]. Conversely, patients with higher health literacy may question the oncologist's decision [6], which may affect their trust. Thus, these findings reflect the tool's sensitivity to the interplay of trust, vulnerability, and empowerment.

### 4.1 Limitations

This study provides evidence for the psychometric properties of the AM‐TiOS‐SF, but some limitations remain. The sample size was adequate, yet recruitment was limited to a single oncological center, recommending replications in other settings.

Although the AM‐TiOS‐SF showed strong internal consistency and structural validity, the absence of test–retest reliability restricts conclusions about the tool's stability over time. However, assessing test–retest reliability is highly recommended to capture the temporal stability of the tool. This scarcity happened due to participants' inability to complete the questionnaires in subsequent chemotherapy sessions and follow‐up visits. Non‐responses have occurred through incomplete questionnaires, failure to respond to follow‐up calls, and participants providing incorrect appointment dates, which made repeated measurements impractical. Of the 197 total participants who completed the first round survey, all were contacted for retest; however, only 27 participants were provided even impartial data, which was insufficient for analysis. Nevertheless, in the absence of test–retest reliability, stability of the tool across time duration and disease stage cannot be drawn. Future studies should therefore employ longitudinal designs with independent samples to establish the temporal stability of the AM‐TiOS‐SF.

### 4.2 Clinical Implications

The validation of the AM‐TiOS among cervical cancer patients is essential, as it enables oncologists to measure the trust level of their patients and quickly identify areas of communication that need improvement. Further, the validated short form (AM‐TiOS‐SF) can help in guiding patient‐centered intervention, which in turn would strengthen the practice of SDM and therapeutic relationships in the clinical care of cancer. In clinical practice, it may serve as a quality indicator for gynecologic oncology services. Further, it may help in designing training programs that are aimed at enhancing communication skills of oncologists in patient–physician communication.

## 5 Conclusions

The AM‐TiOS‐SF demonstrated adequate psychometric properties, consistent with previous studies, and depicted validity in the current sample of cervical cancer patients. Although the findings are promising, recommendations for its use should be made cautiously. Generalizability to the non‐study area populations should be made after further investigations. In research, the AM‐TiOS‐SF can benefit in understanding trust and its predictors, but further study in various settings is needed to assure its large applicability and stability over time and across patient groups.

## Author Contributions

**Sosina W. Tilahun:** conceptualization, methodology, software, data curation, investigation, formal analysis, writing – original draft. **Adiam Nega:** investigation, formal analysis, project administration. **Lealem Wagaw:** investigation, formal analysis, project administration. **Marij A. Hillen:** validation, visualization, writing – review and editing. **Adamu Addissie:** funding acquisition, visualization, validation, writing – review and editing, resources, supervision.

## Funding

The Science for African Foundation to the Developing Excellence in Leadership, Training, and Science in Africa (DELTA Africa) program (Del‐22‐008) supported the fieldwork of the study. S.W.T. completed this thesis project in partial fulfillment of the Master of Public Health in Health Research Ethics at the College of Health Sciences of Addis Ababa University.

## Ethics Statement

This study adhered to the guidelines of the Declaration of Helsinki. Ethical clearance was obtained from the Research Ethics Committee of the Department of Epidemiology and Biostatistics, School of Public Health, Addis Ababa University (protocol number 001, approved January 22, 2025).

## Consent

All participants received information, had the opportunity to ask questions, and signed informed consent after fully understanding the risks and the benefits of participation. Data were collected during waiting periods before therapy or consultation sessions to ensure a calm environment. Two independent data collectors, both with master's training in clinical oncology nursing and unaffiliated with the study site, conducted the data collection. Confidentiality was maintained by anonymizing responses and replacing personal identifiers with codes.

## Conflicts of Interest

The authors declare no conflicts of interest.

## Data Availability

The data that support the findings of this study are available from the corresponding author upon reasonable request.
